# Building a trustworthy AI differential diagnosis application for Crohn’s disease and intestinal tuberculosis

**DOI:** 10.1186/s12911-023-02257-6

**Published:** 2023-08-15

**Authors:** Keming Lu, Yuanren Tong, Si Yu, Yucong Lin, Yingyun Yang, Hui Xu, Yue Li, Sheng Yu

**Affiliations:** 1 Department of Automation, Tsinghua University, Beijing, 100084 China; 2 Department of Gastroenterology, Peking Union Medical College Hospital, Chinese Academy of Medical Sciences and Peking Union Medical College, Beijing, 100730 China; 3 Center for Statistical Science, Tsinghua University, Beijing, 100084 China; 4 Department of Industrial Engineering, Tsinghua University, Beijing, 100084 China

**Keywords:** Neural network, Integrated gradients, Knowledge distillation, Crohn’s disease, Intestinal tuberculosis

## Abstract

**Background:**

Differentiating between Crohn’s disease (CD) and intestinal tuberculosis (ITB) with endoscopy is challenging. We aim to perform more accurate endoscopic diagnosis between CD and ITB by building a trustworthy AI differential diagnosis application.

**Methods:**

A total of 1271 electronic health record (EHR) patients who had undergone colonoscopies at Peking Union Medical College Hospital (PUMCH) and were clinically diagnosed with CD (*n* = 875) or ITB (*n* = 396) were used in this study. We build a workflow to make diagnoses with EHRs and mine differential diagnosis features; this involves finetuning the pretrained language models, distilling them into a light and efficient TextCNN model, interpreting the neural network and selecting differential attribution features, and then adopting manual feature checking and carrying out debias training.

**Results:**

The accuracy of debiased TextCNN on differential diagnosis between CD and ITB is 0.83 (CR F1: 0.87, ITB F1: 0.77), which is the best among the baselines. On the noisy validation set, its accuracy was 0.70 (CR F1: 0.87, ITB: 0.69), which was significantly higher than that of models without debias. We also find that the debiased model more easily mines the diagnostically significant features. The debiased TextCNN unearthed 39 diagnostic features in the form of phrases, 17 of which were key diagnostic features recognized by the guidelines.

**Conclusion:**

We build a trustworthy AI differential diagnosis application for differentiating between CD and ITB focusing on accuracy, interpretability and robustness. The classifiers perform well, and the features which had statistical significance were in agreement with clinical guidelines.

**Supplementary Information:**

The online version contains supplementary material available at 10.1186/s12911-023-02257-6.

## Background

Crohn’s disease (CD) is a chronic and idiopathic inflammatory disease that usually has a disease course with repeating remission-relapses. Intestinal tuberculosis (ITB) is an infectious intestinal disease caused by *Mycobacterium tuberculosis*. The treatment, progression, and prognosis of CD and ITB are different, and the initial correct diagnosis and differentiation between CD and ITB are of critical importance.

Although make different diagnosis between CD and ITB relies on multi-dimension analysis of different examinations (e.g. endoscopy, medical history, radiological findings, molecular tests such as PCR/NGS) endoscopy is an important and essential examination for a timely and accurate diagnosis and is always conducted first [1]. However, the differential diagnosis between CD and ITB can be challenging because the two diseases have a very similar endoscopic appearance. Therefore, diagnosis relies heavily on the experience of the clinician who conducts the examination. This situation often causes incorrect endoscopic diagnosis and results in delayed treatment.

This study aims to facilitate correct interpretation of endoscopic reports and differentiation between CD and ITB using natural language processing. Furthermore, we aim to provide a workflow for obtaining trustworthy neural network classifiers using texts, particularly unstructured texts, such as electronic health records (EHRs). We define a trustworthy neural network as a neural network that can be explained with human understandable phrase features that allow doctors to understand how the model reaches a certain conclusion.

Artificial intelligence (AI) is widely used in the medical field and has been applied to differentiate CD and ITB. However, as the model becomes increasingly complex, the inability of AI users to interpret the decision process has become problematic. Classical AI models, such as support vector machines, random forests and neural networks, are commonly described as “black boxes” due to the lack of interpretability. The interpretability of the AI model in the medical field is an important metric for the following reasons: 1) clinicians should be able to judge if the prediction of the model is reasonable; 2) new interpretable features found by the model can be further verified through clinical studies so that guidelines of the disease can be updated; and 3) clinicians are professionally conservative, and an interpretable model will be more readily accepted than a black-box model.

Recently, research on explanation methods in deep learning has emerged. The integrated gradient (IG) method has the property of being model agnostic and can be derived everywhere for the model parameters. Compared with other methods, the computational cost of IG is relatively small, and therefore it is selected as the interpretation method in our work. Sundararajan et al. [2] show the explanatory effect of IG in the fields of text classification and question answering. In addition, because IG has a small computational cost and derivability in all cases, it is also used to integrate prior knowledge or to correct bias as described by Liu et al. [3] The attribution method represented by IG often means that it can obtain interpretability at the token level, which is still challenging to understand. Chen et al. [4] and Singh et al. [5] proposed a hierarchical interpretation method based on contextual decomposition to solve this problem. They obtained the interpretability of the model for features of different scales. All of these works inspire us to build an interpretable deep learning AI diagnosis system. However, all of the results in the previous works are based on a corpus in English. Few methods and experiments focus on interpreting neural networks with IG in Chinese corpora.

Several works also use neural networks to explain or obtain medical concepts in the medical image processing field. Graziani et al. [6] propose a framework that shifts the attribution focus from pixel values to user-defined images. Experts can explain and trust the network output by checking whether specific diagnostic measures are present in the learned representations. Hu et al. [7] construct a diagnosis model for COVID-19 with CT images and weakly supervised lesion localization with IG. Preuer et al. [8] employed IG to identify the most relevant components of a compound for network prediction of molecular properties and bioactivities. Lauritsen et al. [9] present the Xai EWS—an explainable AI early warning score (EWS) system for predicting acute critical illness using EHRs. Sayres et al. [10] investigate the effect of 2 types of visualization models to indicate diabetic retinopathy scores and expansion heatmaps on the accuracy, speed, and confidence of readers. However, there are few works on building a trustworthy diagnosis application with text data.

### Present work

**Fig. 1:**
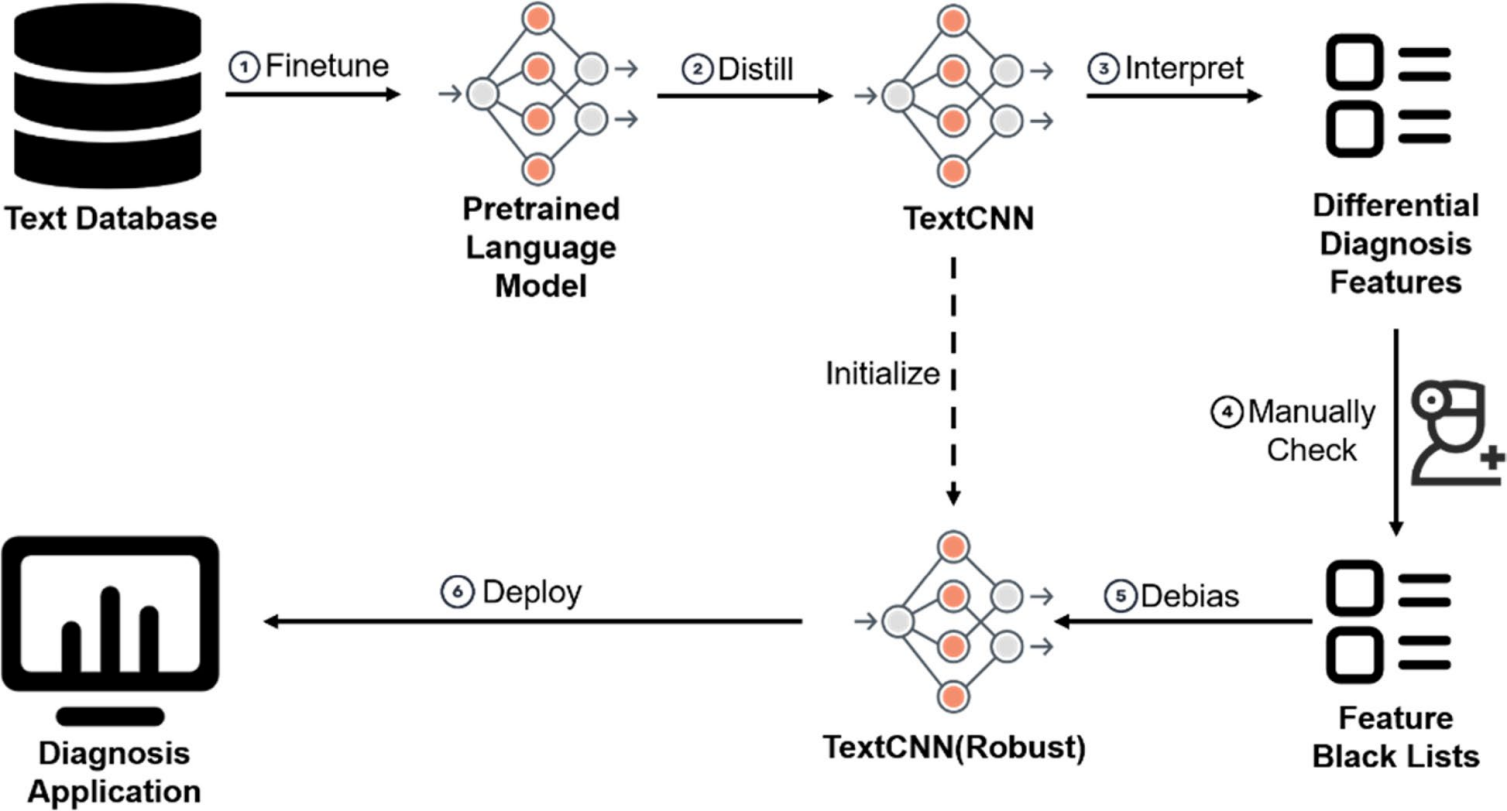
The workflow of building a text-based trustworthy diagnosis model

We introduce a workflow to build a trustworthy AI differential diagnosis system for Crohn’s disease and intestinal tuberculosis. And we also analyze significant diagnostic features we mined. Figure 1 illustrates the whole process of the proposed workflow. From our perspective, a trustworthy AI diagnosis system should have the properties of correctness, interpretability, and robustness. More specifically, correctness means that the classifier is expected to have acceptable accuracy in differential diagnosis; interpretability indicates that doctors know how the classifier works to achieve the diagnosis; robustness indicates that the classifier should not overfit meaningless features in the data and is expected to be mining features with medical significance. This work proposes a 6-step workflow to build a trustworthy differential diagnosis system for Crohn’s disease and intestinal tuberculosis:

1. Finetune**.** In the first step, we finetune a pretrained language model with text description as a classification problem.
2. Distill. We distill the finetuned pretrained language model into a TextCNN model.
3. Interpret. We use Integrated Gradients method to obtain local interpretation of all samples. Then, hierarchical phrase features are selected and filtered by statistical significance as differential diagnosis features.
4. Manually Check. Medical doctors label the differential diagnosis features with medical guidelines and professional knowledge. A set of features that are meaningless or apparent are selected into a blacklist.
5. Debias. We do a debias training by adding an attribution penalty to the loss function. After debias training, the TextCNN model has zero attributes on meaningless features in the blacklist.
6. Deploy. Finally, we deploy this model as a web service. Doctors can query with text descriptions and obtain classification results and visualization of attribution.

To summarize, this study aims to make endoscopic diagnosis of CD and ITB more accurate with the help of natural language processing (NLP) and statistical analysis and builds a trustworthy diagnosis application. The novelty of this workflow is that it employs high precision neural networks and cutting-edge interpretation methods to significantly reduce workloads of clinicians in human-in-loop data mining. Clinicians can only check features instead of predictions to debias the model and make it provide trustworthy results. The workflow can improve the diagnostic accuracy between CD and ITB with fewer risks in clinical application. The codes used in this work are provided on Github.^1^

## Methods

### Notations

We define $D$ as a labeled text dataset with $N$ samples: $D=\{({{\varvec{t}}}_{i},{y}_{i}){\}}_{i=1}^{N}$, where ${{\varvec{t}}}_{i}$ is the token sequence of the i-th endoscopy report. The granularity and the tokenization method are determined by the downstream model. In the pretrained model, the granularity of the token is character-level; ${y}_{i}$ and ${\hat{y}}_{i}$ are the actual and predicted $d$-dimensional one-hot vectors, where $d$ is the number of categories. The model aims to predict ${\hat{y}}_{i}$ from ${{\varvec{t}}}_{i}$ and further obtain a sequence $FT=\{({t}_{i,{b}_{k}},{t}_{i,{b}_{k}+1},\dots ,{t}_{i,{e}_{k}}){\}}_{k=1}^{K}$ that represents the features used by the model when conducting the classification task, and ${b}_{k}$ and ${e}_{k}$ are the start and end indices of the k-th feature. The $FT$ set is important for the differential diagnosis between CD and ITB.

### Methods

This section introduces the development steps of our system. The PTM is first finetuned with labeled training data to obtain a classification model with good diagnostic performance. Then, this large model is distilled into a light TextCNN model. After that, we interpret the distilled TextCNN model with IG and design an analysis method to extract differential attribution features, including hierarchical feature set extraction and a feature selection pipeline.

#### Finetuning pretrained language model

Language model pretraining is an effective approach for improving many natural language processing tasks. RoBERTa-wwm-ext [11] is a state-of-the-art model for conducting text classification in Chinese. This model was trained on Chinese texts with the same architecture of RoBERTa using the whole word masking (wwm) strategy that replaced tokens with mask labels after Chinese tokenization when conducting the masking strategy used in BERT [12]. We chose RoBERTa-wwm-ext for its excellent effect on multiclassification tasks on Chinese text. RoBERTa-wwm-ext can be replaced by other BERT-like models; thus, we refer to RoBERTa-wwm-ext as the pretrained model (PTM) in this article.

The input text is segmented to tokens ${t}_{i}$ by the Chinese word segmentation tool LAC [13]. Special markers are added to ${t}_{i}$ for the PTM, and the input tokens become ${\hat{{\varvec{t}}}}_{i}=\{[CLS],{t}_{i,1},{t}_{i,2},\dots ,{t}_{i,n},[SEP]\}$, where $[CLS]$ and $[SEP]$ are the reserved special tokens for identifying the beginning and end of sentences. For each input text, we use the hidden vector of $[CLS]$ as the embedding of the input. The softmax result after the linear layer was used as the probability for classification:

$${{\varvec{h}}}_{i}^{CLS}=PTM({\hat{{\varvec{t}}}}_{i}),$$

$${{\varvec{p}}}_{i}=softmax\left({\varvec{W}}{{\varvec{h}}}_{i}^{CLS}+{\varvec{b}}\right),$$

where ${{\varvec{h}}}_{i}^{CLS}\in {R}^{{d}_{h}}$ is the representation of the output of the PTM; ${d}_{h}$ is the dimension of the hidden layer; and ${\varvec{W}}$ and ${\varvec{b}}$ are trainable parameters of the linear layer. ${{\varvec{p}}}_{i}\in {R}^{d}$ is the probability for classification. Due to the extremely unbalanced samples in the research, we used the focal loss [14] as the loss function:

$$L=\sum_{i\in D}\sum_{k=1}^{d}-{\alpha }_{k}{y}_{i,k}(1-{p}_{i,k}{)}^{\gamma }log\left({p}_{i,k}\right),$$

where ${\alpha }_{k}$ is the weight of each classification, $\gamma$ is the balance factor, and ${y}_{i,k}$ is the true label.

#### Distilling the PTM into TextCNN

TextCNN is a convolutional neural network for text classification proposed by Kim et al. [15] The input of the model is a sentence, represented as a sequence of word vectors. Let ${{\varvec{x}}}_{i}$ be the word vector corresponding to the i-th word in the sentence with length n. The input can be defined as the concatenation of all of the word vectors:

$${{\varvec{x}}}_{1:n}=\bigcup_{i=1}^{n}{{\varvec{x}}}_{i},$$

where the union symbol denotes vector concatenation and ${{\varvec{x}}}_{1:n}$ denotes the concatenation of the word vectors between the 1st word and the n-th word. A convolution filter matrix ${\varvec{w}}$ is applied to a window of $h$ words to obtain the new feature:

$${c}_{i}=f({\varvec{w}}\cdot {{\varvec{x}}}_{i:i+h-1}+b),$$

where ${c}_{i}$ is a new feature, $b$ is a bias term and $f$ is a nonlinear activation function. This filter is applied to all possible windows in the sentences to obtain a feature list ${\varvec{c}}=[{c}_{1},{c}_{2},\dots ,{c}_{n-h+1}]$. Then, a max pooling operation is employed on this feature list to obtain the feature corresponding to this filter $\widehat{c}=\mathrm{max}({\varvec{c}})$. All max features of various filters are combined as ${\varvec{h}}$, and the logit is obtained with a linear layer:

$$z=f\left({\varvec{W}}\cdot {\varvec{h}}+{\varvec{b}}\right)$$

We distill the finetuned model into TextCNN for two purposes. First, prediction in RoBERTa-wwm-ext is time-consuming and will result in low efficiency. A helpful method is to distill RoBERTa-wwm-ext into TextCNN, which is a significantly faster model. Second, TextCNN is a neural network with word-level features that is easier to interpret. The distillation procedure in our methods follows Hinton et al. [16]. We use ${f}_{t}$ and ${f}_{s}$ to denote the PTM model and the TextCNN model, respectively. Logits of each sample are first calculated according to:

$${{\varvec{z}}}_{t}={f}_{t}\left({{\varvec{x}}}_{i}\right) , {{\varvec{z}}}_{s}={f}_{s}\left({{\varvec{x}}}_{i}\right),$$

where ${{\varvec{x}}}_{i}$ denotes the i-th sample in the training set. Then, a Kullback–Leibler divergence between the softmax logits of the teacher and student models is calculated as the distillation loss function:

$$L={T}^{2}\sum_{i}\frac{\mathrm{exp}\left(\frac{{{\varvec{z}}}_{t}}{T}\right)}{\sum_{j}\mathrm{exp}\left(\frac{{{\varvec{z}}}_{t}}{T}\right)}log\left(\frac{\mathrm{exp}\left(\frac{{{\varvec{z}}}_{s}}{T}\right)}{\sum_{j}\mathrm{exp}\left(\frac{{{\varvec{z}}}_{s}}{T}\right)}\right),$$

where $T$ is a temperature constant. The training loss may also include classification loss as the hard labels, but we only use the distillation loss since this knowledge distillation loss achieves better performance in our work.

#### Differential attribution analysis

Differential attribution analysis aims to identify understandable N-gram features that have significant differences in attribution between different diseases. These differential attribution features are the differential diagnosis features of the neural network models.

**Figure:**
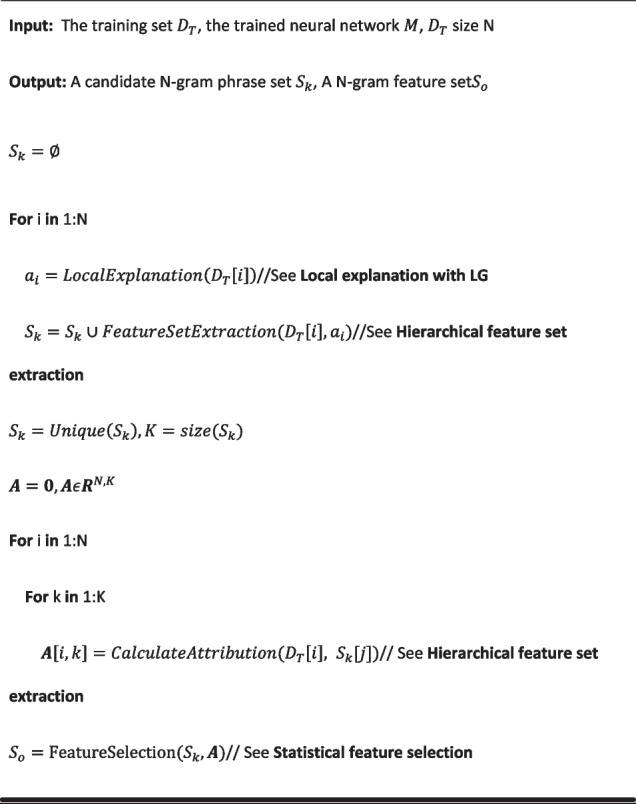
**Algorithm 1** Differential attribution analysis

##### Local explanation with IG

We calculate the attribution of input with the IG method to identify the most important features for classification. IG is an attribution method for neural networks. Attributions are contributions of inputs to the prediction. Formally, suppose a function $F:{R}^{n}\to [\mathrm{0,1}]$ represents the classification function of the PTM, and the token embedding of the input is denoted as ${H}_{i}=({h}_{i,1},\dots ,{h}_{i,l})\in {R}^{l\times {d}_{e}}$. ${d}_{e}$ is the dim of token embeddings. An attribution of the prediction at input ${H}_{i}$ relative to a baseline input ${H}{\prime}$ is a vector ${A}_{F}({H}_{i},{H}{\prime})=({a}_{i,1},\dots ,{a}_{i,l})\in {R}^{l\times {d}_{e}}$, where ${a}_{i,k}$ is the contribution of ${h}_{i,k}$ to the prediction $F(x)$. In our work, we use token embedding of the padding token as the reference baseline input. The IG method conforms to the two axioms of attribution methods namely sensitivity and implementation invariance of the gradient, requires no modification on the neural network architecture and is simple to implement. Therefore, we choose IG as the attribution method in this work.

##### Hierarchical feature set extraction

Words and N-gram phrases are more explainable to humans than individual Chinese characters. Therefore, after obtaining the attributions of the input character tokens, we further derive a hierarchical feature set of words and phrases along with their attributions. Denoting the sample as a Chinese character sequence $t=\{{t}_{1},\dots ,{t}_{l}\}$ with attributions $a=\{{a}_{1},\dots ,{a}_{l}\}$, we can segment this sequence with Chinese word segmentation and obtain word-level tokens $w=\{{w}_{1},\dots ,{w}_{m}\}$ with attributions ${a}_{w}=\{{a}_{w1},\dots ,{a}_{wm}\}$, which are calculated by ${a}_{wi}=\sum_{{t}_{j}\in {w}_{i}}{a}_{j}$. Then, we form a set of phrases $p=\{\{{w}_{p{1}_{1}},\dots ,{w}_{p{1}_{k}}\},\dots ,\{{w}_{p{n}_{1}},\dots ,{w}_{p{n}_{k}}\}\}$ that are successive words whose attribution ${w}_{p{i}_{j}}$ is larger than the $0.9$ quantile of ${a}_{w}$. Then, N-grams (up to 3 words) are generated from each phrase in $p$. The feature set will be the union of N-gram sets obtained from each sample. This N-gram feature set is the set of candidates for differential features.

**Fig. 2:**
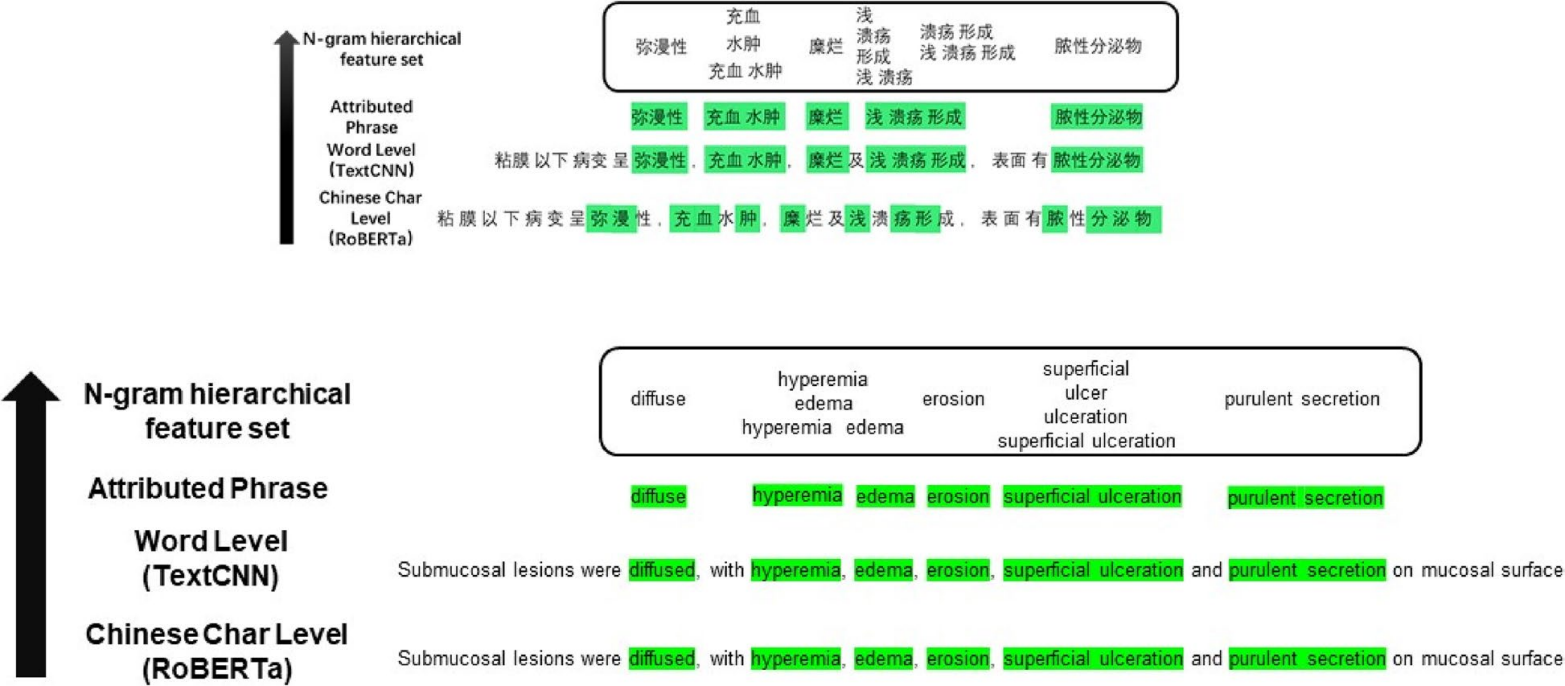
A case demonstration of hierarchical feature set extraction: words or characters in the sentence with positive attribution scores are highlighted with a green background. The extraction process constructs an N-gram hierarchical feature set from bottom (word or character level) to top

After collecting the set of N-gram candidate features, the attributions of each feature in all training samples are calculated and arranged as an attribution matrix $A\in {R}^{N\times K}$, where $N$ is the size of the training set and $K$ is the number of candidate features (Fig. 2).

##### Statistical feature selection

After obtaining the hierarchical feature set and calculating the attribution matrix ${\varvec{A}}$, we further analyze this matrix and perform feature selection to obtain the differential diagnosis features. A feature can be represented by an attribution vector ${{\varvec{a}}}_{k}\in {R}^{1\times N}$ in the attribution matrix. The i-th value in ${{\varvec{a}}}_{k}$ is the attribution of feature $k$ in the i-th sample. We rank the variance of ${\{{{\varvec{a}}}_{k}\}}_{k=1}^{K}$ and select 50 features with the largest variance.

Then, we use a t-test to further select the features with significantly different attributes between the classes. We denote the class labels as $C$. When selecting a feature that is highly attributed in the samples of class $c$ and shows relevantly low attribution in other classes, a t-test is employed to calculate the statistical significance. Let $I(c)$ represent the index set of samples with class $c$. The t statistic is calculated as

$${t}_{k}=\frac{\frac{1}{\left|I(c)\right|}\sum_{j\in I(c)}{a}_{j}-\frac{1}{n-\left|I(c)\right|}\sum_{j\notin I(c)}{a}_{j}}{\sqrt{\frac{\sum_{j\in I(c)}{({a}_{j}-\frac{1}{\left|I(c)\right|}\sum_{j\in I(c)}{a}_{j})}^{2}}{{\left|I(c)\right|}^{2}}+\frac{\sum_{j\notin I(c)}{({a}_{j}-\frac{1}{n-\left|I(c)\right|}\sum_{j\notin I(c)}{a}_{j})}^{2}}{n-{\left|I(c)\right|}^{2}}}}$$

The *p* value ${p}_{k},k=\mathrm{1,2},\dots ,K$ can be obtained for each feature, and we rank *p* values as ${p}_{1}\le {p}_{2}\le \dots \le {p}_{K}$. Since this is a multiple comparison, we employed the Benjamini–Hochberg method to control the false discovery rate (FDR) at 0.01. This adjustment begins with ${q}_{K}={p}_{K}$ and sequentially calculates ${q}_{k}$ from the largest index by the following rules:

$${q}_{k}=\left\{\begin{array}{ccc}{p}_{k}\times \frac{m}{k}& ,& {p}_{k}\times \frac{m}{k}\le {q}_{k+1}\\ {q}_{k+1}& ,& {p}_{k}\times \frac{m}{k}\ge {q}_{k+1}\\ 1& ,& {p}_{k}\times \frac{m}{k}\ge 1\end{array}\right.,\mathrm{ k}=1, 2, \dots ,\mathrm{ K}$$

The features with ${q}_{k}\le 0.01$ will be selected and ranked by difference $d=\frac{1}{\left|I(c)\right|}\sum_{j\in I(c)}{a}_{j}-\frac{1}{n-\left|I(c)\right|}\sum_{j\notin I(c)}{a}_{j}$. These features are differential attribution features of class $c$.

#### Debias finetuning by attribution penalty

Differential attribution analysis proposes a method to identify readable diagnosis features that the neural networks rely on. However, we find that the features extracted by the above methods indicate that neural networks make classifications with inappropriate and unwanted features. Therefore, we include a debias finetuning processing in our workflow that allows clinical doctors to adjust model performance using their professional knowledge.

First, a blacklist of unwanted features of each disease is manually selected from differential attribution features. For example, a blacklist containing disease names themselves is as follows.

$$Blacklist=\{CD:[Crohn^{\prime}s\ disease],\ ITB:[intestinal\ tuberculosis]\}$$

The above blacklist means that when the model classifies a real CD sample in fine tuning, the word “Crohn’s disease” is expected to be a neutral feature. To achieve that, we add an attribution penalty to the classification loss and fine tune the model.

$$Loss=\frac{1}{n}\sum_{i=1}^{n}\mathrm{FocalLoss}({y}_{i},{\hat{y}}_{i})+\lambda \times \frac{1}{{l}_{i}}\sum_{j=1}^{{l}_{i}}{({a}_{ij}- targe{t}_{ij})}^{2}$$

$\lambda$ is a hyperparameter determined by cross-validation, and ${l}_{i}$ denotes the length of the i-th sentence in the token. ${target}_{ij}$ is defined as a tokenwise label. If a token is included in the blacklist, this label equals 0. Otherwise, ${target}_{ij}$ equals the attribution of this token. The attribution penalty will lead the model to ignore the blacklisted tokens during classification.

$$targe{t}_{ij}=\left\{\begin{array}{ccc}{a}_{ij}& ,& toke{n}_{ij}\notin Blacklist[{y}_{i}]\\ 0& ,& toke{n}_{ij}\in Blacklist[{y}_{i}]\end{array}\right.$$

## Data and experimental setup

### Dataset

A total of 1271 electronic health records of successive patients who had undergone colonoscopies at Peking Union Medical College Hospital (PUMCH) and were clinically diagnosed with CD (*n* = 875) or ITB (*n* = 396) from January 2008 to November 2018 were included in this study. Research approval was obtained from Peking Union Medical College Hospital’s Ethics Committee (approval no. S-K894). All the patients had given informed consent. We separated 80% of the data into the training set and 20% of the data into the test set for training models and analysis.

The clinical diagnoses of CD were made via endoscopic results, medical history, pathological features, and treatment follow-up based on the Chinese consensus of IBD (2018) by IBD specialists in this hospital. The clinical diagnoses of ITB were confirmed by the presence of at least one criterion from the following: 1) positive acid-fast bacilli on histological examination, 2) positive *M. tuberculosis* culture, 3) radiologically or colonoscopically proven TB, and 4) full response to anti-TB therapy. Colonoscopies were performed with Olympus CF-Q260 or H260 colonoscopes and were conducted by well-trained endoscopists at PUMCH. Based on the well-established terminology used by endoscopists to describe colonoscopic images, we extracted descriptions of colonoscopic images of the patients’ index colonoscopy in the form of free text. Clinically confirmed diagnoses extracted from the hospital information system (HIS) were used as labels (Table 1).

**Table 1:** Some examples of the collected and analyzed samples

Text sample		Diagnosis
Chinese description	钩拉法循腔插镜至回盲部。回盲部巨大不规则溃疡, 周边结节样隆起, 回盲瓣显示不清, 局部活检6块, 质硬, 送病理及抗酸染色; 余所见结肠、直肠粘膜光滑, 血管纹理清晰, 无充血、糜烂、溃疡及新生物。	CD
Translation	The colonoscope was introduced into the rectum and advanced to the terminal ileum using the Pull method. Large irregular ulcer(s) in the terminal ileum, with peripheral nodule(s). The ileocecal valve was not well seen. Biopsy of 6 pieces, which were firm, for pathological investigation and acid-fast stain test. Other findings: smooth colorectal mucosa, normal vascular pattern, no hemorrhage, no erosion and ulcer, no neoplasm	
Chinese description	肠道准备欠佳循腔进镜至回肠末段约15 cm,进镜顺利, 末段回肠粘膜可见多发溃疡, 形态欠规则, 约0.5–1.5 cm大小, 中心凹陷, 周边粘膜肿胀隆起, 表覆灰白苔, 取活检共3块, 质韧。回盲瓣呈唇形,阑尾开口看不清楚,所见全结肠、直肠粘膜光滑,血管纹理清,半月襞完整,未见糜烂、溃疡及新生物。	ITB
Translation	Poor bowel preparation. The colonoscope was introduced into the rectum and advanced to 15 cm from terminal ileum. Multiple cratered ulcers of 0.5–1.5 cm in the mucosa of terminal ileum, with peripheral edematous mucosa, covered by gray and white fur. Biopsy of 3 pieces, which were tough. Lip-shaped ileocecal valve. The vermix opening was not well seen. Findings: smooth colorectal mucosa, normal vascular pattern, normal semilunar folds, no erosion and ulcer, no neoplasm	

## Results

Table 2 displays the classification performance of the various models. The standard dataset refers to the original data. The distilled TextCNN gave the highest overall accuracy of 0.84 and the highest F1 score of CD of 0.88. By contrast, the standard TextCNN obtained the lowest overall accuracy of 0.81, which is 3 percentage points lower than that of distilled TextCNN. The Robust TextCNN gave the highest recall rate of 0.87 in CD and the highest F1 score of 0.77 in ITB. PTM did not show advantages in any task. In the noisy dataset, the distilled TextCNN performed poorly, with an overall accuracy of 0.50. The Robust TextCNN thoroughly outperformed the distilled TextCNN that gave an overall accuracy of 0.70.

**Table 2:** Classification results between CD and ITB

Dataset	Model	CD			ITB			Overall Accuracy
		precision	recall	F1	Precision	recall	F1	
Standard	TextCNN	**0.92**	0.81	0.86	0.62	0.81	0.70	0.81
	PTM	0.87	0.86	0.87	0.75	0.77	0.76	0.83
	TextCNN(distill)	**0.92**	0.84	**0.88**	0.70	**0.83**	0.76	**0.84**
	TextCNN(Robust)	0.87	**0.87**	0.87	**0.77**	0.77	**0.77**	0.83
Noisy	TextCNN(distill)	0.60	0.61	0.61	0.33	0.32	0.32	0.50
	TextCNN(Robust)	0.82	0.83	0.87	0.83	0.71	0.69	0.70

Table 3 shows the differential diagnosis features from each model. For CD, all the classifiers gave *ulcer, linear*, and *anastomosis*. Notably, only robust TextCNN gave the feature *cobblestone-like* that was unique and set as a specific diagnostic feature in CD. Other features found by the classifiers included *hyperemic, edematous*, and *stenosis*. In addition, PTM gave much fewer features than the other three classifiers. For ITB, all four models gave similar features, including *ileocecal valve, polyp*, and *remain opened*. PTM model found *protruding lesions*, while the Robust TextCNN model found *round lesions*.

**Table 3:** Differential diagnosis features selected by the attribution analysis (both original terms displayed in Chinese and their translation) are listed. Features supported by clinical guidance are in bold

Model	CD	ITB
TextCNN	循腔 进镜 至 The colonoscope was introduced into the rectum and advanced to 进镜 至 advanced to **溃疡** **ulcer** **纵行** **linear** **吻合口** **anastomosis** 可见 findings 循腔 进镜 The colonoscope was introduced into the rectum 进镜 The colonoscope was introduced 进镜 至 回肠 advanced to ileum 至 to **充血** **hyperemic** **糜烂 溃疡** **erosion and ulcer** 距 肛门 xx cm from anus 至 回肠 末段 to terminal ileum **纵行 溃疡** **linear ulcer** 克罗恩病 Crohn’s disease 乙状结肠 sigmoid colon 至 回肠 to ileum 肛门 anus 溃疡 及 ulcer and **水肿** **edematous** **狭窄** **stenosis**	**回盲瓣** **ileocecal valve** 盲肠 Cecum 盲袋 pouch 余 other 余 所见 other **息肉** **polyps** **回盲瓣 变形** **ileocecal valve deformity** 回盲瓣 呈 ileocecal valve 取 活检 biopsy 检查所见 findings 阑尾 开口 vermix opening 循腔 进 镜达 The colonoscope was introduced into the rectum and advanced to **盲袋 结构** **pouch** 取 tissue submitted 呈 was 病理 pathological **变形** **deformity** 未见异常 normal 进 镜达 The colonoscope was introduced into the rectum and advanced to 送 病理 biopsy from **皱襞 光 整** **smooth folds** 活检 4块 biopsy of 4 pieces
TextCNN (distill)	循腔 进镜 至 The colonoscope was introduced into the rectum and advanced to **纵行** **linear** 进镜 至 advanced to **吻合口** **anastomosis** 克罗恩病 Crohn’s disease 可见 findings **溃疡** **ulcer** 循腔 进镜 The colonoscope was introduced into the rectum and advanced to 距 肛门 xx cm from anus **纵行 溃疡** **linear ulcer** 乙状结肠 sigmoid colon 进镜 The colonoscope was introduced into the rectum and advanced to 进镜 至 回肠 advanced to ileum **水肿** **edematous** 至 回肠 末段 to terminal ileum 肛门 anus **充血** **hyperemic** **糜烂 溃疡** **erosion ulcer** 克罗恩病 治疗后 after treatment for Crohn’s disease 至 回肠 to ileum 克罗恩病 治疗后 复查 reexamination after treatment for Crohn’s disease 降结肠 乙状结肠 descending colon and sigmoid colon 散 在 diffuse	**回盲瓣** **ileocecal valve** 盲肠 cecum 余 other 盲袋 pouch 余 所见 other **息肉** **polyps** 检查所见 findings 取 活检 biopsy 回盲瓣 呈 ileocecal valve was 腔 进 镜达 The colonoscope was introduced into the rectum and advanced to 取 tissue submitted **回盲瓣 变形** **ileocecal valve deformity** 进 镜达 The colonoscope was introduced into the rectum and advanced to 呈 was **皱襞 光 整** **smooth folds** 病理 pathological 皱襞 fold **变形** **deformity** **环形** **round** 阑尾 开口 vermix opening **皱襞 光** **smooth folds** 未见异常 normal **持续 开放** **remain opened**
PTM	**溃疡** **ulcer** 克罗恩病 Crohn’s disease **吻合口** **anastomosis** 克罗恩病 治疗后 after treatment for Crohn’s disease **纵行 溃疡** **linear ulcer** 肛门 anus 距 肛门 xx cm from anus 肛门 口 anus	块 piece **隆起** **protruding lesions** 循腔 The colonoscope was introduced into the rectum and advanced to 改变 lesion **息肉样 隆起** **polyps-like protruding lesions** 回盲瓣 ileocecal valve 样 改变 lesion 样 隆起 protruding lesions 活检 1 块 biopsy of 1 piece 1 块 1 piece 余 所见 other 至 to 回盲瓣 呈 ileocecal valve was 至 回肠 末段 to terminal ileum 软 soft 阑尾 开口 vermix opening **光 整** **smooth** **糜烂** **erosion** 至 回肠 to ileum 质 软 soft 活检 4块 biopsy of 4 pieces 3 块 3 pieces
TextCNN (Robust)	**纵行** **linear** **吻合口** **anastomosis** **纵行 溃疡** **linear ulcer** 可见 findings **充血** **hyperemic** 散 在 diffuse **铺路 石样** **cobblestone-like** **水肿** **edematous** **狭窄** **stenosis** 距 肛门 xx cm from anus **轻度 充血** **moderately hyperemic** 治疗后 复查 reexamination after treatment 轻度 moderate 肠道准备 bowel preparation 局部 localized **铺路 石样 改变** **cobblestone-like** 复查 reexamination **小溃疡** **small ulcer** 乙状结肠 sigmoid colon	回盲瓣 ileocecal valve 回盲瓣 呈 ileocecal valve was 取 活检 biopsy **回盲瓣 变形** **ileocecal valve deformity** 检查所见 findings 盲肠 cecum **息肉** **polyps** 余 other **环形** **round** 余 所见 other 活检 4块 biopsy of 4 pieces 取 活检 4块 biopsy of 4 pieces 循腔 进 镜达 The colonoscope was introduced into the rectum and advanced to **持续 开放** **remain opened** 4块 4 pieces **皱襞** **fold** 进 镜达 advanced to **瘢痕形成** **scarring**

To be noticed, the terms shown in Table 3 are those that computationally contributed to the classification, but they alone are not decisive. For example, in the model, the occurrence of polyps contributes positively to classifying as ITB, but the final prediction could be either ITB or CD according to the presence of other features. Indeed, real diagnosis should also consider other examinations and lab tests, as differential diagnosis between CD and ITB is very difficult. The terms in boldface are clinically meaningful ones (the rest involve computational noise) and can be used to highlight the text input to provide supporting evidence for the prediction, thus adding interpretability to the model. These highlighted terms can also guide inexperience physicians to pay attention to key features that may help the differentiation.

## Discussion

Corresponding to the definition of trustworthy AI we proposed before, we discuss the contributions of this work from three aspects: accuracy, interpretability and robustness. For each aspect, we analyzed our contributions both from the perspective of the techniques and the perspective of clinical medicine.

In addition, we would like to further emphasize that our method is task-agnostic, which means it can be generalized to other challenging differential diagnosis tasks taking free text as input. Although our model cannot solve the differential diagnosis between CD and ITB solely based on free-text, it could potentially being used as an auxiliary tool for clinicians.

### Accuracy of differential diagnosis

For clinical medicine, this research provided a new possible approach for differentiating CD and ITB. Differential diagnosis of CD and ITB has long been a challenging and essential problem. Retrospective Chinese studies show that approximately 65% of CD patients have been misdiagnosed with ITB at least once [17]. At the same time, another study indicated that more than 40% of CD patients had received tentative anti-TB treatments due to ambiguous diagnoses. Traditional histologic or pathologic evidence, such as caseating granuloma or positive acid-fasting staining, was considered to be the gold standard with high specificity. However, these examinations are time-consuming and have a sensitivity lower than 50%. Thus, an immediate differential diagnosis with high sensitivity and specificity is valuable.

The four classifiers all achieved an overall accuracy above 80%, demonstrating that artificial intelligence can provide satisfactory results in clinical practice. This could help clinicians, particularly for inexperienced patients, to make a more accurate diagnosis. The distilled TextCNN and robust TextCNN provided a balanced precision and recall rate, which was also crucial for clinical practice.

It is important to note that knowledge distillation leveraged the language knowledge of PTM and obtained a higher classification accuracy. As shown in Table 2, the overall accuracy of PTM was 2% higher than that of TextCNN. Then, distilled TextCNN achieved an even higher overall accuracy (by 1%) than PTM, and its F1 scores of both diseases also ranked first. In addition, the student model is significantly lighter than the teacher model. Therefore, knowledge distillation contributed to obtaining a better model while requiring less training and deployment resources. These advantages make diagnosis models more conducive to deployment and adoption.

### Interpretability

Our previous study built a classifier for classifying CD and ITB using a convolutional neuron network (CNN) [18]. However, due to the low interpretability of CNN, the previous classifier could not explain the basis of the diagnosis to doctors, greatly limiting its clinical application (Fig. 4 in Additional file 1 illustrate the difference between a black box model and an interpretable model). This research solved the previous problem. The front end can clearly show the classification result and the supporting details, based on which clinicians can make further judgments.

### Robustness

Debias training is an essential component of this system. First, it provides an effective method for doctors to customize the diagnosis model with their knowledge. In addition, debias training restricts the model from attributing the classification results to meaningless or unreasonable features in the blacklist and achieves significantly better results than the baseline model on the noisy dataset. Although we restricted the model from learning certain significant features in the standard dataset, it still reached the same level of accuracy as the models without debias training. The optimization of deep neural networks would by default exploit and extract any feature whose distribution in the training data correlates with the class label, and the extracted features are not guaranteed to be informative. Manually labeling the feature blacklist and penalizing it during training adds an additional regularization to the optimization of the neural network, forcing it to avoid unreasonable features in the blacklist to find features that truly differentiate and diagnose the two diseases.

The differential features for classification found by the classifiers were highly consistent with the guidelines. We noticed that Robust TextCNN provided more specific features, such as cobblestone appearance, while TextCNN and distilled TextCNN tended to offer more general features. This may occur because patients with these specific features comprise only a small portion of the total data set. TextCNN and distilled TextCNN tended to ignore these features due to the small sample size and corresponding low statistical power. However, Robust TextCNN gave these specific features, most likely due to the penalty coefficient of the general features. Therefore, in the noisy dataset, Robust TextCNN strongly outperformed distilled TextCNN. A further discussion of Robust TextCNN is given below. In summary, clinicians can use diagnostic evidence from different classifiers to support their judgment.

However, we should note that some patients may not be distinguished purely by endoscopy and need further examinations due to the similarity of the endoscopic results of CD and ITB. Therefore, additional clinical and biological research on CD and ITB may be conducted to evaluate whether feature extraction by AI can help improve the upper limit value of the accuracy while differentiating CD and ITB.

### Limitations

Our current work is limited in that it only uses the description text of endoscopy reports. It should be noted that a loss of information can occur when inexperienced clinicians describe the endoscopic findings, and there are also CD and ITB cases that are not distinguishable by endoscopy. Therefore, a combination of other clinical lab examinations(e.g. acid-fast staining, PCR based methods) and the text model can potentially improve the model’s classification capability and requires further research. Additionally, language patterns may differ across institutions. Although the extracted differential features appear consistent with clinical experience and guidelines, the portability of the text model at different institutions requires further testing.

## Conclusion

In this work, we developed a differential diagnosis application using state-of-the-art natural language processing for differentiating between CD and ITB, focusing on the accuracy, interpretability, and robustness aspects of a trustworthy AI. The resulting classifier performed well, and the extracted differential features that met statistical significance conformed with clinical guidelines, proving the effectiveness of our human-in-circle workflow.

### Supplementary Information

**Additional file 1. **

## Data Availability

Clinical data cannot be published due to ethical consideration. Please contact the corresponding author for potential access of clinical data.

## Abbreviations

CD: Crohn’s disease
ITB: Intestinal tuberculosis
HER: Electronic health record
PUMCH: Peking Union Medical College Hospital
PCR: Polymerase chain reaction
NGS: Next generation sequencing
AI: Artificial intelligence
IG: Integrated gradient
EWS: Early warning score
NLP: Natural language processing
wwm: Whole word masking
PTM: Pretrained model
HIS: Hospital information system
CNN: Convolutional neuron network

## Footnotes

1 https://github.com/Lukeming-tsinghua/Interpretable-NN-for-IBD-diagnosis
