# Supplementary material for: Building a trustworthy AI differential diagnosis application for Crohn’s disease and intestinal tuberculosis
Source: BMC Med Inform Decis Mak. 2023 Aug 15;23:160. doi: 10.1186/s12911-023-02257-6 (PMC10426047; doi:10.1186/s12911-023-02257-6)
Supplement: Supplementary file 1 — Additional file 1. [file 12911_2023_2257_MOESM1_ESM.docx]

**Appendix**

**A.1 Training and analysis setup**

**Finetuning the PTM.** PTM *Chinese-RoBERTa-wwm-ext was used for this study*. We used its tokenizer to tokenize the input text and fine tune the model with batch size of $16$ and learning rate of $5\times{10}^{-5}$ on an NVIDIA GeForce RTX 3090 GPU. We used cross-entropy loss and the AdamW optimizer with weight decay on the parameters except for the biases and weights of layer normalization during the training.

**Training TextCNN with knowledge distillation.** We employed TextCNN with the same structures as those used Kim et al.^[6]^. The widths of the filters ranged from 2 to 5, and there were 100 filters for each size. The Chinese word segmentation tool *LAC* was used to segment input sentences and to build the vocabulary. Sentences that were shorter than 256 were padded, and longer sentences were cut to the same length. The dimension of word embedding was 100, and all embeddings were initialized randomly. We first trained a TextCNN on true labels with Adam as the optimizer and focal loss as the loss function. The learning rate in this step was 0.001. The learning rate in knowledge distillation was 0.001, the weight of Kullback–Leibler divergence loss was 0.2, and the temperature was 2.

**Differential diagnosis features analysis.** We used integrated gradients to calculate the attributions of all of the samples. First, we created the top 30,000 N-gram features by selecting word combinations from the vocabulary with the highest frequency. The parameter ranged from 1 to 3. For each sample in the training dataset, we calculate the attribution score for these 30,000 n-gram features obtained by hierarchical feature set extraction. Then, we further selected the top 500 features with the highest variance.

**Building the noisy test set.** To further evaluate the debias performance, we built a noisy test set. After extracting differential diagnosis features and manually creating the blacklist, we created the noisy test set by adding CD features in the blacklist to the beginning of ITB samples and vice versa. If the features in the blacklist were not attributed, then the model would obtain better performance than that before debias training, and the performance should be close to that on the standard test set. Among these 500 features, we performed multiple t-tests with the Benjamini-Hochberg correction for each feature, and we justified whether a feature was significant for differential diagnosis by checking whether their p values were under $1\times{10}^{-7}$. If the mean attribution of ITB of a feature is higher than that of CD, we assign it as a significant differential diagnosis feature for ITB and vice versa.

**Model debias.** Uninformative features were manually selected into the black list. During debias training, the TextCNN model was initialized with parameters after knowledge distillation. Training losses included focal loss for classification and mean square error loss for the attribution penalty. The weight of the Focal Loss was $1\times{10}^{-4}$. The optimizer was Adam with the learning rate of $5\times{10}^{-3}$.

**A.2 Visualization of extracted features with heat maps**

The heat maps in Figure 3 visualize the features used by the classifiers. The hues in the figures represent the attributions of each feature to the final classification result. We found that TextCNN, Robust TextCNN, and distilled TextCNN showed separated clear zones and dark zones, while PTM did not show separated zones in CD.

|  |  |
| --- | --- |
|  |  |


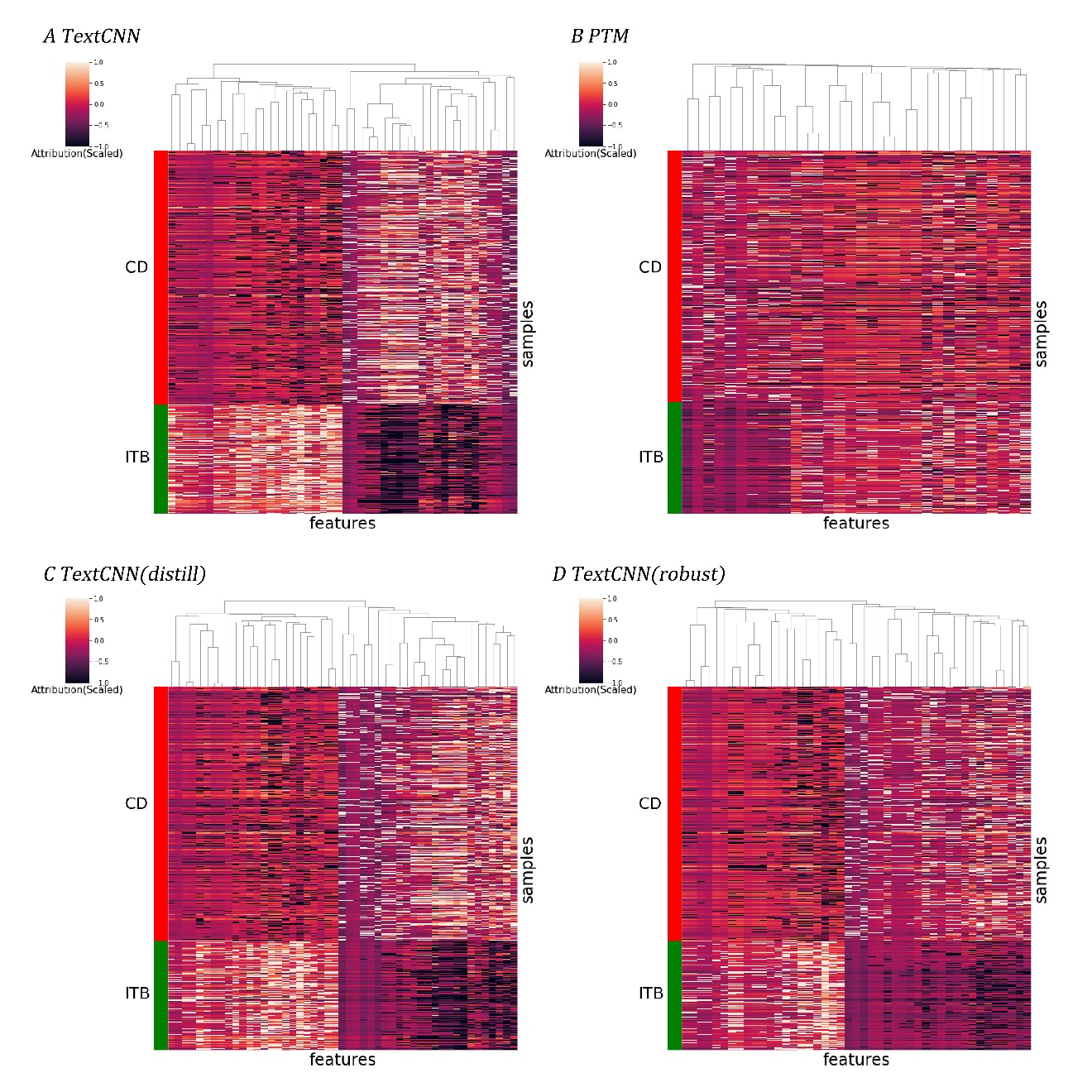


Figure 3. Cluster heat maps showing attribution differences between two diseases: hierarchical clustering separates differential diagnosis features that we mined into two groups, visually demonstrating that these features contribute to the differential diagnosis between two diseases.


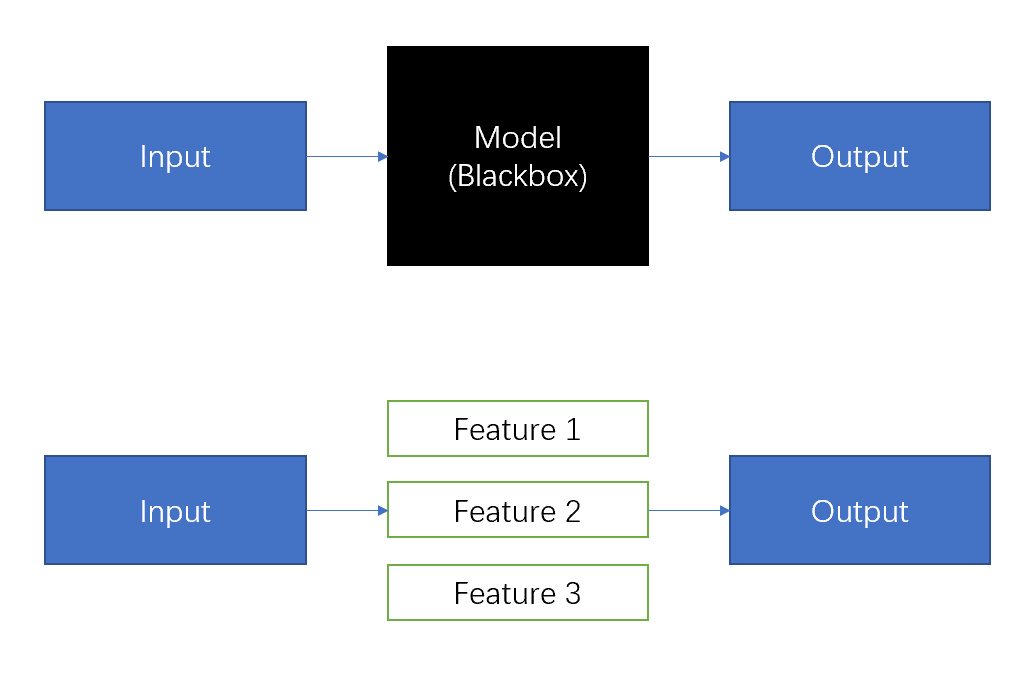


Figure 4. The difference between a black box model and an interpretable model. An interpretable model could give the feature used during the diagnosis, which is very helpful to doctors^[1]^.

[1] Barnett AJ, Schwartz FR, Tao C et al. A case-based interpretable deep learning model for classification of mass lesions in digital mammography. Nat Mach Intell. 2021;3:1061–1070. doi: 10.1038/s42256-021-00423-x
